# Supplementary material for: Genotypic and Phenotypic Characteristics of Moraxella catarrhalis from Patients and Healthy Asymptomatic Participants among Preschool Children
Source: Pathogens. 2022 Aug 29;11(9):984. doi: 10.3390/pathogens11090984 (PMC9503219; doi:10.3390/pathogens11090984)
Supplement: Supplementary file 1 [file pathogens-11-00984-s001.zip › pathogens-1855727-supplementary.pdf]

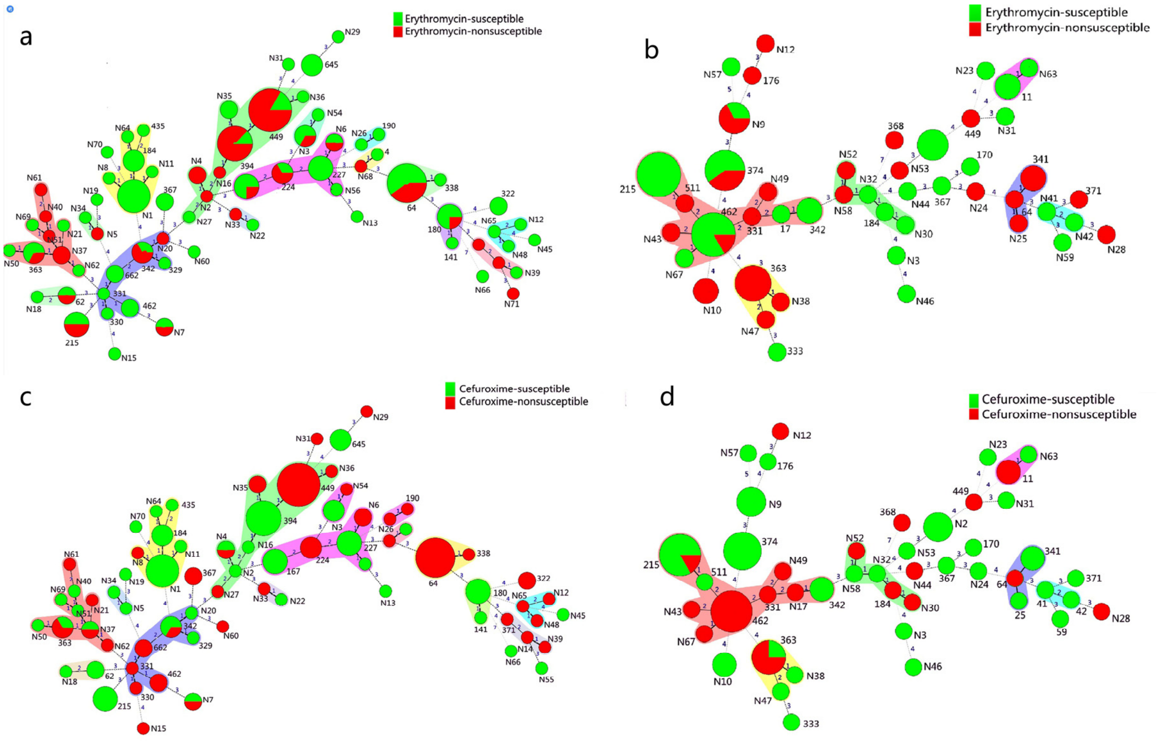

Figure S1. Susceptibility or non-susceptibility to *M. catarrhalis* to erythromycin and cefuroxime based on allelic profiles of MLST. (**a**) Different colors indicate the susceptibility or non-susceptibility of patients to erythromycin; (**b**) Different colors indicate the susceptibility or non-susceptibility of healthy asymptomatic participants to erythromycin; (**c**) Different colors indicate the susceptibility or non-susceptibility of patients to cefuroxime; and (**d**) Different colors indicate the susceptibility or non-susceptibility of healthy asymptomatic participants to cefuroxime.
